# Evaluation of Feasibility and Acceptability of a Text-Messaging Intervention for Tobacco Cessation in India

**DOI:** 10.1093/ntr/ntad163

**Published:** 2023-08-28

**Authors:** Abhijit Nadkarni, Leena Gaikwad, Miriam Sequeira, Richard Velleman, Joseline D'souza, Ankita Hoble, Rajanish Haldankar, Pratima Murthy, Felix Naughton

**Affiliations:** Centre for Global Mental Health, Department of Population Health, London School of Hygiene and Tropical Medicine, London, UK; Addictions and Related Research Group (ARG), Sangath, India; Addictions and Related Research Group (ARG), Sangath, India; Addictions and Related Research Group (ARG), Sangath, India; Addictions and Related Research Group (ARG), Sangath, India; Department of Psychology, University of Bath, Bath, UK; Addictions and Related Research Group (ARG), Sangath, India; Addictions and Related Research Group (ARG), Sangath, India; Addictions and Related Research Group (ARG), Sangath, India; National Institute of Mental Health and Neurosciences, Bengaluru, India; School of Health Sciences, University of East Anglia, Norwich, UK

## Abstract

**Introduction:**

The aim of our study was to assess the feasibility and acceptability of a brief behavioral intervention for tobacco cessation delivered via mobile phone text messaging in India.

**Aims and Methods:**

We conducted an uncontrolled intervention cohort study in adult current users of tobacco. The participants received intervention messages on their mobile phones for eight weeks. We collected qualitative data about participants’ perceptions of intervention delivery and receipt, acceptability, and feasibility of the intervention. The outcomes measured at 3 months post-recruitment were self-reported 7- and 28-day point-prevalence abstinence, and Alcohol, Smoking, and Substance Involvement Screening Test (ASSIST) risk categories for tobacco—low (0–3), moderate (4–26), and high (≥27).

**Results:**

We recruited 26 eligible participants, and 22 completed the outcome assessments. The participants generally perceived the intervention content to be simple to access and useful in facilitating a change in tobacco use. None of the participants indicated that they wanted to discontinue receiving the intervention messages. Some suggestions for enhancing acceptability included supplementing text messaging with more intensive counseling and the use of multimedia content. Eighteen percent of participants reported abstinence in the past 7 and 28 days. A greater proportion of those who used smokeless tobacco were abstinent at follow-up compared to those who smoked (42.9% vs. 6.7%; *p* = .04).

**Conclusions:**

If effective, simple and low-cost mobile phone text messaging can be used to deliver interventions for tobacco use, and has the potential to be scaled up so it can be delivered to populations of smokers interested in receiving cessation support.

**Implications:**

Our study is an important step towards the development of a contextually relevant intervention suited for low- and middle-income countries and which is responsive to the needs of both those who use smoked and smokeless tobacco. If found to be effective, our intervention would be a scalable solution to overcome the human resource related barrier to accessing tobacco cessation services in low resource settings.

## Introduction

Globally, tobacco use (both smoked and smokeless) is associated with high mortality and morbidity including cancers, and respiratory and cardiovascular diseases. In 2015, smoking was ranked among the five leading risk factors by disability-adjusted life years (DALYs) and responsible for 11.5% of global deaths (6.4 million), of which 52% were in four countries (China, India, United States, and Russia).^1^ In 2017, 2.5 million DALYs and 90 791 lives were lost across the globe due to cancers attributed to smokeless tobacco (SLT), and 6 million DALYs and 258 006 lives were lost from ischemic heart disease attributed to SLT.^2^ More than 85% of the disease burden due to SLT was in South and Southeast Asia, with India accounting for 70%.^2^ The overall burden of “smoking only,” “smokeless only,” and “dual use” of tobacco in India is 7.2%, 17.9%, and 3.4%, respectively, with the prevalence in males being greater than in females.^3^ Despite the high burden related to tobacco use, the treatment gap for access to tobacco cessation services in India is almost 92% (ie, 9 out of 10 of those who use tobacco have no access to cessation services)^4^ due to poor availability and accessibility of tobacco cessation services, inequitable distribution of resources, and nonavailability of culturally relevant and contextual interventions.^5,6^

Brief Interventions are cost-effective in reducing substance use including alcohol and tobacco.^6^ However, implementation at scale especially in low- and middle-income countries (LMICs) faces several barriers such as lack of financial and structural resources, limited or nonexistent training and support, and high workload of primary care workers, limiting their potential to deliver BI.^7,8^

Hence, nonresource-intensive interventions are needed to increase the penetration and coverage of tobacco cessation interventions in low health resource settings.^5^ The use of mobile technologies for health care delivery provides such an opportunity even in the least developed countries, as these are easily scalable, and sustainable.^9^ The increasing availability of low-cost mobile devices in LMICs such as India, thus provides a unique opportunity to increase access to low intensity interventions.

Although there is evidence of the effectiveness of text-messaging interventions for tobacco cessation, this evidence comes primarily from high income countries (HICs),^10^ and the limited evidence from LMICs has methodological shortcomings and limited generalisability.^11^ Additionally, the evidence from HICs is not easily transferable to LMICs as patterns of tobacco use, health beliefs associated with tobacco use, and awareness of associated health risks are influenced substantially by context and culture. Finally, no text-messaging interventions have been developed or tested with those who use SLT.

This study aimed to overcome the accessibility hurdles related to conventional health care delivery (eg, shortage and inequitable distribution of cessation services) by developing and evaluating a simple, evidence-based intervention for those who use tobacco that can be delivered using low-cost and easily available mobile text messaging. This paper describes an investigation into the acceptability and feasibility, and the preliminary impact, of the ToQuit intervention, which was built through a systematic intervention development methodology (a key characteristic that differentiates ToQuit from the existing mCessation program in India).

## Methods

### Study Design

Uncontrolled intervention cohort study with pre- and post-outcome evaluation and nested qualitative study.

### Participants

We included adults (≥18 years) of any gender who used tobacco (smoked and SLT), residing in India, who were willing to quit tobacco, and who owned a personal mobile phone that could receive text messages. Individuals using tobacco who were enrolled in other tobacco cessation programs at the time of screening, or who were unable to read and reply to messages in English or Hindi language were excluded from this study.

Participant recruitment advertisements were shared online via our organization’s social media accounts and posters were displayed at health facilities, village local administration offices, and other public places in Goa. Potential participants willing to quit tobacco were asked to send an SMS with the text “TOQUIT” to a designated mobile number. After receiving the message from a potential participant, researchers called them back to screen for eligibility. Verbal informed consent was recorded from eligible respondents to participate in the study. Non-consenting respondents were sent a link to access an e-flyer on the benefits of quitting tobacco. We stopped recruitment upon identifying the pre-determined sample of 30 eligible participants required to meet the objectives of the study. As our study aimed at contributing to the treatment development through the assessment of the acceptability and feasibility of the intervention, no formal sample size estimations were carried out. The sample size for the treatment cohort was based on our experience with previous intervention development projects,^12,13^ feasibility of recruitment, and adequacy to meet study objectives.

### Baseline Data

We collected baseline data over the phone and included sociodemographic details, tobacco use patterns, the Alcohol, Smoking, and Substance Involvement Screening Test (ASSIST), and intervention message delivery preferences (preferred language, days, and time) from the consenting participants. ASSIST is a screening tool used to detect psychoactive substance use and related problems,^14^ validated for use in India.^15^ While the ASSIST has questions related to alcohol, tobacco, and other substances, for the purpose of this study, we only administered the six questions related to the use of tobacco. The total score for the tobacco-related questions can range from 0 to 31, with a higher score indicating greater risk.

### Intervention

ToQuit is a text-messaging-based low-intensity digital intervention for smoked and SLT cessation, developed and adapted to the Indian context through formative research informed by the Medical Research Council framework for developing and evaluating complex interventions.^16^ The intervention development process included four sequential steps. In step 1, we used a recent (<2 years old), relevant (mobile-based tobacco cessation intervention), and high-quality (robust and reproducible methodology) systematic review to identify the core components of effective tobacco cessation interventions. In step 2, we conducted in-depth interviews (IDIs) with tobacco cessation experts and users of tobacco to understand the patterns of tobacco use in the local context, examine the content and form of the intervention for perceived utility, and explore and define treatment expectations and desired outcomes for tobacco users; and used the data from these interviews to define intervention content and delivery. In step 3, we surveyed international experts to elicit information on the acceptability, feasibility, and perceived effectiveness of each of the intervention strategies identified through steps 1 and 2. Finally, in step 4, we conducted participatory intervention development workshops with contextually embedded tobacco cessation practitioners to develop the conceptual framework of the ToQuit intervention using the outputs of the preceding steps.

The intervention messages covered topics such as psychoeducation about the consequences of tobacco use and the benefits of quitting; goal setting; managing goals and self-monitoring of behavior; tobacco avoidance strategies; self-awareness and reflection; how to seek social and pharmacological support; identifying and managing cravings; and relapse prevention. Participants received 3–6 messages each day for 3–4 days a week for a total of 8 weeks. All participants received all the messages and there was no customization based on individual characteristics; and all the messages were pull messages, that is, not requiring any responses from the participants. These decisions were made based on our experience of developing and delivering a text-messaging intervention for hazardous drinking.^17^

The mobile numbers of recruited participants were shared with the technology partner which had an automated system to send the intervention messages as per the delivery preferences of participants, and shared weekly message delivery reports with the research team for monitoring.

### Smoking Outcome Assessment

The outcomes of interest measured at 3-months post-recruitment were (1) self-reported 7- and 28-day point-prevalence abstinence, and (2) risk categories based on the ASSIST score—low (0–3), moderate (4–26), and high (≥27). The outcome tools were administered by trained researchers over the phone.

### Feasibility/Acceptability Assessment

We also conducted IDIs with a sub-sample of participants to understand their overall experience of the intervention and perception of the feasibility and acceptability of the intervention content and message delivery platform. The IDIs were conducted immediately after the 3-month outcome evaluation. The interview questions were informed by the research objectives of the study and were designed to explore indicators of acceptability and feasibility such as perspectives about the intervention, challenges faced, feedback on the utility of the content of the messages, and perceived impact on their tobacco use. The quantitative and qualitative data were collected over the phone by trained and experienced research assistants.

### Ethics

The project was approved by the Institutional Review Board of the host institution.

### Analysis

The qualitative data was analyzed by two independent coders (JD, AH) using Nvivo version 11. Our epistemological position recognizes that many aspects of the acceptability and feasibility of an intervention must be inferred through an examination of the subjective experiences of the individuals receiving the intervention. The transcripts of the interview were read by the two coders to immerse themselves in the data. Subsequently, they generated preliminary codes and grouped them into themes and sub-themes based on the original research questions and sections in the topic guide. After finalizing the initial codebook, they coded the remaining transcripts and generated higher order themes and sub-themes. Finally, they developed a narrative interpretation of the themes and selected relevant illustrative quotes. Both the coders had direct experience with the study setting, but neither of them was directly involved with the intervention development. The perspectives of the wider research team which contributed to the project in various capacities facilitated the interpretation of the findings as related to a priori theoretical and empirical foundations of complex intervention development and testing in the study settings. The quantitative data was analyzed using STATA SE 16. Descriptive analyses were conducted using *t* test and chi-square tests for means and proportions respectively. Since we did not want to use the small dataset for any inferential analyses, we did not use any advanced strategies such as imputation of missing data.

## Results

We received the TOQUIT SMS from 47 potential participants. Of these, nine did not respond to our follow-up call and five were seeking help for their tobacco using family member. Of the remaining 33 (70%), one each was ineligible as they were not residing in India, did not own a personal phone, and could not read Hindi/English. Of the 30 eligible participants, 26 (87%) consented to participate in the study. Reasons for refusal of consent included not having time, no interest in the study, and difficulty in understanding the terms of the study.

The baseline sociodemographic characteristics of the participants in the case series and the nested qualitative study are shown in Table 1. A majority of the participants were male, educated, employed, and married. A majority of the participants smoked tobacco and had a moderate risk score on the ASSIST.

**Table 1.** Sample Characteristics

Variable	Quantitative study *N* = 26 *N* (%)	Completed follow-up *N* = 22 *N* (%)	Qualitative study *N* = 17 *N* (%)
Mean age (SD), range	38.2 (12.7), 18–66	36.6 (12.5), 18–66	38.2 (12.9), 20–66
Gender			
Male	24 (92.3)	20 (90.9)	15 (88.2)
Female	2 (7.7)	2 (9.1)	2 (11.8)
Education status			
Completed secondary	2 (7.7)	2 (9.1)	2 (11.8)
Completed higher secondary	8 (30.8)	5 (22.7)	4 (23.5)
Graduate	11 (42.3)	11 (50.0)	8 (47.1)
Post-graduate	5 (19.2)	4 (18.2)	3 (17.7)
Employment status			
Employed	18 (69.2)	15 (68.2)	11 (64.7)
Unemployed	1 (3.9)	1 (4.6)	1 (5.9)
Student	3 (11.5)	3 (13.6)	2 (11.8)
Retired	3 (11.5)	2 (9.1)	2 (11.8)
Homemaker	1 (3.9)	1 (4.6)	1 (5.9)
Marital status			
Single	11 (42.3)	11 (50.0)	9 (52.9)
Married	15 (57.7)	11 (50.0)	8 (47.1)
Type of tobacco			
Smoked	16 (61.5)	15 (68.2)	13 (76.5)
Smokeless	10 (38.5)	7 (31.8)	4 (23.5)
ASSIST tobacco risk level			
Moderate risk (score 4–26)	21 (80.8)	19 (86.4)	14 (82.4)
High risk (26 and above)	5 (19.2)	3 (13.6)	3 (17.7)

 Twenty-two (85%) participants completed the follow-up at 3 months, of which 17 (77%) consented to participate in the qualitative study. There were no statistically significant differences between those who completed outcome evaluation and those who did not on baseline sociodemographic factors, type of tobacco used, ASSIST score, and ASSIST tobacco risk level. None of the participants requested for the intervention delivery to be discontinued i.e., none of the participants sent us a “STOP” text message indicating that they wanted to stop receiving the intervention messages.

### Feasibility and Acceptability of the Intervention

#### Overall Experience

Most participants reported that the messages, especially related to the negative health effects, helped them realize that their tobacco use was problematic (Table 2). Some mentioned waiting with eager anticipation for the messages as they were *“interesting and motivating*.” The confidentiality provided by private messages was appreciated as they could access the intervention without the knowledge of others. As one of the participants stated:

**Table 2.** Feasibility and Acceptability of the Intervention

Overall experience	• Increased awareness that tobacco use was problematic • Interesting and motivating • Confidentiality was appreciated
Intervention content	• Meaningful • Relevant • Encouraging • Appropriate • Comprehensible • Meaningful
Participant learnings	• Raised awareness about ◦ Ill effects of tobacco ◦ Reasons for consumption ◦ Steps to manage urges ◦ Management of peer pressure
Feasibility of SMS platform	• Comfortable receiving messages via SMS • Preference for advanced platforms eg, Whatsapp
Delivery	• Appreciated customization of time of delivery based on participant preference • Three to four messages per day preferred
Barriers	• Language of the messages • Generic nature of the messages • Lack of personalization • Limited details about skills such as strategies to quit and craving management

*…this programme of yours is like a path to follow silently. It tells you that you are not alone, someone is there with you. You go ahead and we will support you. You just have to walk ahead with confidence.* (Smoker, M, 20Y)

#### Intervention Content

The participants felt that the intervention messages were meaningful, relevant and encouraged them to quit.

*“I had a good experience because everything was for my benefit. I use* [tobacco] *paste, it has no benefits, there are only ill effects. The messages told me it does not have any good effect. So, these messages were good for me.”* (SLT user, F, 54Y)

Many participants stated that in general, the messages were appropriate, comprehensible, and meaningful even for those with a basic education. Most of the participants said that the messages were appropriate in length and that they did not want messages longer than 5–6 lines on their phones. One participant thought that the length of the messages would not matter as long as the information was interesting.

*It all depends on the content. If the content is valuable then I don’t mind however long the message is. It can be even one paragraph. That doesn’t matter. But if you are just going to send me things that I know for the sake of sending me messages…like I need to stop smoking and all. Like… those messages, if long, are of no use…* (SLT user, M, 34Y)

#### Participant Learnings From the Intervention

A few participants mentioned that the messages raised their awareness about the ill effects of tobacco, reasons for consumption, steps to be taken when one gets the urge to consume tobacco, and ways to deal with friends who consume tobacco. One participant mentioned that he realized that he was *“destroying his health for just five rupees”* instead of buying food which could nourish his body. Two participants mentioned that they did not learn anything from the intervention.

#### Feasibility of SMS Platform

A few participants stated that they were very comfortable receiving messages via SMS and that they had read all the messages they received. On the other hand, many participants stated that they would have preferred receiving the same messages over WhatsApp instead of SMS. This was for two reasons: one, they rarely pay attention to SMS messages because of the many promotional messages they receive via that feature, and two, because more engaging content such as videos and images can be sent via WhatsApp. Two participants said that phone calls would be more feasible and impactful compared to SMS messages.

#### Delivery of Messages

Most participants said that they were happy with the time at which they received the messages as it was sent according to their preference. There was a range of suggestions with regard to the scheduled delivery time of the messages—some suggested sending the messages early in the morning so that the user is reminded about their goal as soon as they wake up, and others preferred evening slots as it allowed them to read the messages at leisure. A few opined that it was best to personalize the scheduled delivery of messages to suit the individual’s tobacco consumption pattern. On this note, one participant said that those who smoke heavily need constant support since they get the urge to smoke at around breakfast time, during lunch, and in the evening and would prefer messages to be sent at these time periods.

*Time, technically as early as possible. Because when you wake up and if you see it the first thing, it will have a lot more impact than if you see it when you have already smoked three - four cigarettes. Then the impact will be a lot less.* (Smoker, M, 33Y)

Most participants thought that messages sent daily at three to four times a day would make the intervention more effective while some preferred alternate days. One participant liked the frequency with which he received the messages, however, he suggested an extended duration of the intervention for at least 6 months to a year.

### Enablers and Barriers in Engaging With the Intervention

Very few participants were able to specify what helped them engage with the intervention messages and the subsequent impact on their tobacco use. One participant who failed to quit tobacco use reported that the language used in the messages was a barrier to engaging with the messages and felt that simpler words would have been more impactful. Other reported barriers to engagement were the generic nature of the messages, lack of personalization, and limited details about skills such as strategies to quit, and craving management.

*See, your content, your messages were like motivating… They were telling me why I should quit but they were not telling me how I should quit.* (Smoker, M, 33Y)

#### Effect of the Intervention on Tobacco Use

A few participants reported that the intervention messages had a significant role in controlling their tobacco use and urges as the messages were relevant to them and that helped them to control their tobacco use.

*Yes, madam there was one change. I had stopped tobacco completely for twenty to twenty-five days. I have not taken even cigarette and gutkha* (type of SLT) *also. Nothing in those days. I feel like you* (ToQuit) *have contributed to this* (abstinence). (Smoker, M, 23Y)

One of the participants reported that he completely stopped consuming tobacco after 8 days of receiving the messages. Another reported a slight change in their consumption behavior and that the interval between consumption increased. Another reported that the messages informing him about the toxic substances present in tobacco products were helpful when he got the urge to consume because they made him think of harmless substitutes instead.

*I read your messages and then I started thinking what can happen* (due to tobacco use) *and what are the reasons for that, and what I can do about it. Then around the third and fourth week, your messages were related to how you can avoid the urge to smoke. So, I researched a little bit about what I can do. I came to know that I can listen to music or can do some exercise, I can keep myself busy with that. I started to go out a little bit…Then I started getting results like… yes, I can avoid it. I mean, in between I got the urge to smoke but now I am thinking about something else.* (Smoker, M, 20Y)

Another participant who was motivated to quit found that the messages were not effective enough to help her quit and sought pharmacotherapy instead.

*Your messages are strong, but I don’t think they will help me -- because I am addicted for very long, so I do not feel like getting rid of it … I was planning to go to my family doctor to ask whether there are any medicines because I want to get rid of it.* (SLT, F, 54Y)

#### Suggestions for Improvements in the Intervention

Many participants suggested that the intervention messages should be supplemented with either telephone or online counseling sessions or medicines as they thought that SMS alone had a weaker impact on their tobacco consumption. Some suggested that adding links to relevant videos and success stories would make the messages more engaging as these create a greater impact on motivation to quit. Some of the participants wanted the option to reply to the messages. One participant explained that responding to messages and phone calls can be helpful because some people feel lonely during the quitting process, and responding to messages would make quitting tobacco easier and quicker.

### Impact on Tobacco Use

Of those who completed the outcome evaluation, four (18.2%) reported abstinence both in the past 7 and 28 days. At baseline, 21 (80.8%) participants were moderate risk and 5 (19.2%) were high risk. At 3 months, 1 (4.6%) was low risk, 20 (90.9%) were moderate risk, and 1 (4.56%) was high risk. A significantly greater proportion of SLT users were abstinent at follow-up compared to smokers (42.9% vs. 6.7%; *p* = .04).

## Discussion

This paper presents the acceptability, feasibility, and preliminary impact testing of a text-messaging tobacco cessation intervention that we developed using a systematic intervention development methodology. Our process data indicated that it is feasible to identify and recruit patients using both online and offline dissemination methods. The change in the tobacco use outcomes was in the right direction, and overall, the intervention was perceived to be useful by the participants.

There is substantial evidence that automated text-messaging smoking cessation interventions independently or as a supplement to routine smoking cessation support is superior to smoking cessation support alone in achieving higher quit rates.^10^ However, unlike our intervention, these interventions are focused only on smoked tobacco. Additionally, evidence of such interventions in LMICs is limited, with most evaluations conducted only in upper middle-income countries in urban areas.^11^ Finally, none of our potential participants had to be excluded because they were enrolled in other cessation programs, indicating the poor uptake of existing interventions. Hence, our study is an important step toward the development of a contextually relevant intervention suited for LMICs and responsive to the needs of both smoked and SLT users.

An important observation was about ownership of and comfort with the use of a mobile phone, a key consideration for the feasibility and acceptability of ToQuit. While only one potential participant was ineligible because they did not own a phone, those who received the intervention reported being comfortable using the relevant functionalities on their phone. These were important observations because, despite the high, and increasing, tele-density in India, there still remain concerns about the actual coverage (because many phones are shared across a family or other group), and proficiency of mobile phone owners with the various functionalities of their phones.^18^

One important recommendation by the participants was to use more advanced messaging platforms as they were more commonly used and allowed more advanced functions such as the sharing of videos. As a part of another similar project we had examined options such as WhatsApp, but ultimately decided to continue with text messaging for reasons of scalability (ease of access in rural areas compared to other messaging platforms which require data packages), and concerns around data privacy.^17^ However, these platforms could become preferred options in the future as they become more accessible and secure to use for health care delivery.

There were some limitations to this study. Due to the COVID-19 pandemic restrictions, the abstinence outcome could not be biochemically verified using salivary cotinine estimation as originally planned, and we had to rely on self-reported abstinence. On the other hand, virtual methods allowed us to overcome catchment area boundaries to recruitment, a limitation inherent to screening only in primary care. Alcohol use and common mental disorders such as depression and anxiety commonly influence tobacco cessation efforts and we were not able to examine this as we did not screen for any of those potentially co-existing conditions. One of the key strengths of our study lies in the triangulation of quantitative and qualitative findings, allowing for a more holistic understanding of the feasibility and acceptability of ToQuit. Although the quantitative outcomes of tobacco use were in the desired direction, the absence of a control arm precludes making any conclusion about the role of the intervention in this change. However, the quit rates were similar to the mCessation program in India,^19^ and findings from the qualitative study, especially those related to perceived mechanisms and effect, provide a strong rationale for further effectiveness testing of ToQuit using an appropriate study design.

Some key implications of our findings to future definitive testing of ToQuit include study design characteristics, content of the intervention, and delivery platform. A mixed recruitment strategy that involves offline screening (eg, in primary care) and online strategies would help overcome concerns around selection bias in the latter. Customization of content delivered based on participant characteristics and allowing for both push- and pull-messages could potentially enhance engagement and impact of the intervention. Finally, more advanced and commonly used messaging platforms such as WhatsApp could be considered for greater reach, provided concerns around confidentiality and safety of data can be suitably addressed.

## Conclusions

The outcome of our study is an understanding of the acceptability and feasibility of a text-messaging intervention for tobacco cessation for delivery in the Indian context. This has been further confirmed in a pilot RCT of the ToQuit intervention (submitted for peer review). As the final step, if found to be cost-effective in a definitive RCT, ToQuit has the potential to address the human resource related barriers to the implementation of tobacco cessation interventions in low resource settings.

## Data Availability

The data that support the findings of this study are available from the corresponding author, AN, upon reasonable request.
